# Genome-Wide Identification of Somatic Aberrations from Paired Normal-Tumor Samples

**DOI:** 10.1371/journal.pone.0087212

**Published:** 2014-01-30

**Authors:** Ao Li, Yuanning Liu, Qihong Zhao, Huanqing Feng, Lyndsay Harris, Minghui Wang

**Affiliations:** 1 Centers for Biomedical Engineering, University of Science and Technology of China, Hefei, China; 2 School of Information Science and Technology, University of Science and Technology of China, Hefei, China; 3 School of Public Health, Anhui Medical University, Hefei, China; 4 Seidman Cancer Center, School of Medicine, Case Western Reserve University, Cleveland, United States of America; Shenzhen Institutes of Advanced Technology, China

## Abstract

Genomic copy number alteration and allelic imbalance are distinct features of cancer cells, and recent advances in the genotyping technology have greatly boosted the research in the cancer genome. However, the complicated nature of tumor usually hampers the dissection of the SNP arrays. In this study, we describe a bioinformatic tool, named GIANT, for genome-wide identification of somatic aberrations from paired normal-tumor samples measured with SNP arrays. By efficiently incorporating genotype information of matched normal sample, it accurately detects different types of aberrations in cancer genome, even for aneuploid tumor samples with severe normal cell contamination. Furthermore, it allows for discovery of recurrent aberrations with critical biological properties in tumorigenesis by using statistical significance test. We demonstrate the superior performance of the proposed method on various datasets including tumor replicate pairs, simulated SNP arrays and dilution series of normal-cancer cell lines. Results show that GIANT has the potential to detect the genomic aberration even when the cancer cell proportion is as low as 5∼10%. Application on a large number of paired tumor samples delivers a genome-wide profile of the statistical significance of the various aberrations, including amplification, deletion and LOH. We believe that GIANT represents a powerful bioinformatic tool for interpreting the complex genomic aberration, and thus assisting both academic study and the clinical treatment of cancer.

## Introduction

Various aberrations including amplification, deletion and translocation of genomic sequence are distinctive features of cancer cells [1], [2]. Frequent genomic aberrations are reported to be related with dysfunction of oncogenes and tumor suppressor genes [1], [3], [4]. Research on genomic aberrations [1], [2], [4]–[12] has greatly revolutionized our understanding of the biological mechanisms that play important roles in tumourigenesis and progression. Associations between patient outcome and genomic aberrations ranging from focal amplification [9], [11] to whole-genome aberration pattern [5] have also been demonstrated in clinical studies. Current technologies for high-throughput profiling of genome-wide aberrations in tumor samples include array comparative hybridization (aCGH) [13], single nucleotide polymorphism genotyping microarray (SNP array) [14] and more recently next-generation sequencing (NGS) [15]–[18]. By allowing for whole-genome analysis of copy number alteration (CNA) and allelic imbalances such as loss of heterozygosity (LOH) with high resolution [19], SNP arrays currently represent an efficient platform with relatively low cost and are particularly suitable for studying a large number of tumor samples.

Due to the unique and complicated nature of tumor, crucial issues have been encountered in analysis of genomic aberrations using SNP array data, including contamination of tumor DNAs by normal stroma or lymph cells admixed in tumor samples [20]–[29], shift of signal baseline occurring in aneuploid tumors [21], [24]–[28], and signal noise associated with local GC content [26], [27], [30]. These issues can largely affect genotyping signals in tumor sample, leading to dramatically altered LRR (log R ratio, representing totally signal intensity) and BAF (B allele frequency, representing the fraction of B allele) signals. A number of computational approaches [21], [23]–[28], [31] have been proposed in order to accurately detect different kinds of aberrations from tumor SNP array data. However, only a few methods can successfully deal with above issues because they usually cannot be addressed separately and therefore dramatically confound interpretation of tumor SNP array data [21], [25]–[28].

In some studies of cancer genomic aberrations [25], [26], [32]–[36], tumors are paired with matched normal samples from the same patient. Although not frequently available, the matched normal samples can be used to further facilitate analysis of tumor samples. Serving as a reference, the genotypes of the paired normal sample can be very helpful in determining the corresponding genotype in tumor and therefore genomic aberrations. Moreover, such information is also decisive in ascertaining whether an aberration found in tumor is somatic (i.e., the corresponding genomic region in paired sample retains normal) or germline (i.e., the corresponding genomic region in paired sample is also altered). Regardless of the advantages mentioned above, genotype information of normal sample is not fully adopted in current methods. For example, whilst ASCAT [25] is one of the “state of the art” approaches, it only uses SNP array data of normal sample to filter out homozygous SNPs with fixed thresholds for genotyping signals. As another efficient method, OncoSNP [26] treats matched normal sample as a “noise” template for removing array-specific noise from tumor sample without taking the genotype information into account. Another concern for current computational methods used in paired SNP array data analysis is that germline variants in matched normal sample are ignored, which instead can be very crucial in quantitatively modelling of tumor genotyping signals and meanwhile provide additional information to discover somatic aberrations in cancer genome.

Whilst great efforts have been made to improve performance in identifying aberrations from tumor SNP array data, methodologies focusing on downstream analysis of genome-wide aberrations remain limited. The GISTIC method [6], [10] provides a promising framework for this purpose, in which statistical significance of aberrant regions is evaluated by permutation test in order to discover recurrent aberrations with critical biological properties in tumor initiation and development. However, the drawback of GISTIC is that raw genotyping signals in tumor SNP array are directly used as measurements of genomic aberrations, which are indeed prone to normal cell contamination, signal baseline shift and other issues discussed previously, since they may dramatically alter genotyping signals in tumor SNP array. To facilitate systematic studies of cancer genome, it is desirable to develop a new approach that evaluates the statistical significance of genomic aberrations identified by SNP array analysis methods and distinguishes recurrent aberrations from random genomic changes.

In this study, we present an efficient bioinformatic tool called GIANT, which is based on the statistical framework of our previous GPHMM method [27]. By incorporating genotype information of matched normal sample, GIANT provides identification of various kinds of somatic/germline aberrations with superior performance, even for aneuploid tumors accompanied with high normal cell contamination. Furthermore, it allows for discovery of statistically significant aberrations when multiple tumor samples are available. We describe a comprehensive evaluation of GIANT using different data sets, with comparison to current computational methods for tumor SNP array data analysis.

## Materials and Methods

### Datasets for Paired Normal-tumor Samples

#### Replicate SNP arrays for breast cancer and normal sample

Replicate pairs of a breast cancer and matched normal sample described in [27] was analyzed with the Human 610-Quad (v1.0) DNA Analysis BeadChip Kits (Illumina Inc., San Diego, CA, USA) with the assistance of the W. M. Keck Foundation Microarray Resource (New Haven, CT, USA). Raw SNP array data obtained from genotyping experiment was pre-processed and analyzed by the Illumina BeadStudio utility. In addition, the genotyping signals were further normalized by tQN to correct possible asymmetry in BAF signals caused by dye bias.

#### Simulated tumor SNP arrays

HapMap sample NA06991 hybridized to the Illumina 550K array was used to generate a simulated tumor SNP array dataset [37]. Ten genomic regions altered with copy number gain/loss and copy neutral LOH were added into the HapMap sample, with the proportion of normal cell contamination ranging from 0% to 100% with an interval of 5%. This data set was downloaded from the website as described in [37].

#### Dilution series of cancer cell-lines

Dilution series of an aneuploid cancer cell line (ATCC: CRL-2324D) was generated by mixing with matched normal cell line (ATCC: CRL-2325D) in 0–0.9 proportion [37]. DNA mixture was then hybridized to Illumina Human370K BeadChips. This data set was downloaded from the NCBI GEO database with accession number: [GEO: GSE11976]. All chromosomes were retained during the analysis.

#### Paired tumor SNP arrays

Four pairs of urothelial tumor and matched normal samples were hybridized to Illumina HumanCNV370 SNP array [37]. This data set was downloaded from the NCBI GEO database with accession number: [GEO: GSE11976]. Another tumor SNP array dataset used in this study include 112 breast cancer samples with matched blood samples, which was downloaded from the website described in [25]. For study of individual tumors, HER2-positive breast cancer samples (case 601) [26], including surgically obtained and micro-dissected tumor material and pure stroma, were downloaded from the NCBI GEO database with accession number: [GEO: GSE23785]. In addition, breast cancer sample 7207, with matched normal sample and a dilution sample (a mixture of 50% tumor and 50% normal sample), was downloaded from the NCBI GEO database with accession number: [GEO: GSE16400].

### Genotype Calls for Paired Normal Samples

In this study, we employed GPHMM [27] to analyze SNP array data of matched normal samples, as it not only allows for germline variant detection but also provides automatic genotype calling for each SNP. The global parameters of GPHMM were set to the default except that the mixture level was fixed to 0 since in this case tested samples consist of pure normal cells. Genotype calls made by GPHMM were based on the maximal conditional probability, which was estimated by the statistical model during training procedure. It should be pointed out that with the existence of germline variants, the genotype obtained from this step is not restricted to diploid genotypes AA, AB or BB. Instead, it can be any possible genotype described in Table S1 in File S1. For further analysis, the genotypes of matched normal sample were uniquely transformed to the corresponding total copy number contributed by both alleles (denoted by 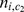) and proportion of B allele (denoted by 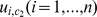), respectively.

### Paired Samples HMM for Tumor SNP Array Data Analysis

In this study, we introduce a novel paired samples hidden Markov model (PSHMM) for statistical modeling and analysis of tumor SNP array data. A comprehensive description of PSHMM is available in the Text S1.

Generally, suppose there are two samples collected in the study: one is a mixed sample (e.g. cancer samples investigated in this study) consisting of two kinds of related cells (denoted as 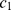, 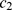) with different genotypes, and the other (here refers to matched normal sample) is a paired sample consisting of only 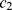. The genotypes of 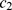 can be explicitly determined from SNP array data for the paired sample, but the genotypes of 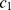 are “hidden” as the genotyping signal of the mixed sample is actually generated from a mixture of DNA from both 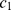
and 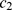 with unknown proportion. The goal is to determine 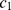
’s genotype information, represented by totally copy number 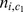
and proportion of B allele 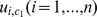, and corresponding genomic aberrations from the SNP array data for the mixed sample, with the aid of genotype information of 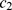 inferred from paired SNP array data. Note that the genotypes of 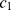 and 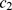 are genetically associated with each other. Here, we assume that the 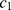
‘s (tumor) genotype can only be homozygous when the 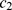
’s (matched normal) genotype is homozygous, and the 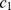
’s genotype can be homozygous or heterozygous when the 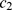
’s genotype is heterozygous.

### Emission Probability

To automatically deconvolute the genotyping signals generated from mixed tumor DNAs, we adopted empirical formulas proposed in [20], in which the relationship of LRR/BAF signals and cancer cell proportion in tumor sample is quantitatively modelled. Specifically, given hidden state *s* in PSHMM (defined in Table S1 in File S1), we can then formulate the emission probability of LRR signal *l_i_* at the *i^th^* probe by using the following normal distribution:
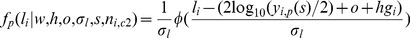
(1)with
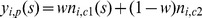
(2)where σl denote the variance of LRR and *w* is the proportion of cancer cells in tumor sample. Note that complicating factors such as tumor aneuploidy and genomic bias associated with local GC contents are taken into account in the emission probability function, with *h* being the unknown coefficient for local GC percentage *g^i^* at the *i^th^* probe and o being the baseline shift of LRR signal. Similarly, we can formulate the emission probability of BAF signal *b^i^* as follow:
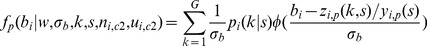
(3)with
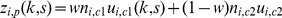
(4)where σb denote the variance of BAF and G is the number of genotypes in state s.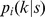 is the prior probability of observing *K^th^* genotype (conditional on s) at the *i^th^* probe in tumor SNP array data.

In practice, genotypes inferred from matched normal sample may contain genotyping errors. In this case, Equation (1) and (3) should be replaced with the formulas that we previously proposed for single tumor SNP array analysis [27], in which the genotypes of 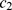 are limited to three normal genotypes, ‘AA’, ‘AB’ and ‘BB’. In addition, to delineate fluctuation in genotyping signals for tumor sample, we use uniform distribution to account for the intrinsic randomness of genotyping errors. Taken together, the full emission probability of PSHMM is formulated as the total probability of observing 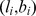 at the *i^th^* probe in tumor SNP array data:
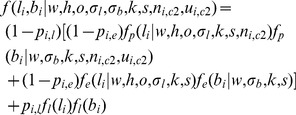
(5)where 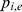 is the probability of genotype error for the *i^th^* probe in normal sample and 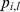 is the probability of genotyping signal fluctuation at the *i^th^* probe in tumor sample.

Taken together, there are five parameters in emission probability functions, including cancer cell proportion *w*, LRR baseline shift *o*, BAF and LRR signal variances *σ_b,_ σ_l,_* and GC coefficient *h*. Since the SNP array data for tumor and matched normal sample may exhibit dramatic difference in these parameters, we estimated the parameters for tumor samples independently from tumor SNP array data by using parameter estimation approach described below.

### EM Algorithm for Parameter Estimation

We used Expectation Maximization (EM) algorithm for HMM training and parameter estimation, which is designed to iteratively find maximum likelihood estimates of parameters associated with hidden latent variables. For the E step in *n^th^* iteration of EM algorithm, expectation of the partial log-likelihood containing emission probability function is formulated as follow:
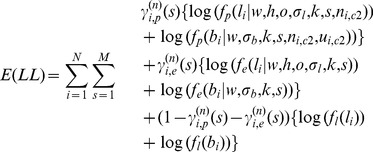
(6)where 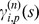 and 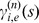
are the conditional probabilities of normal genotype error and genotyping signal fluctuation given hidden state *s*. Next, for the M step we need to find these abovementioned parameters that maximize Equation (6). For example, by taking the partial derivative with respect to *o* and setting it to zero, we can obtain the following formula to update *o* in nth iteration:



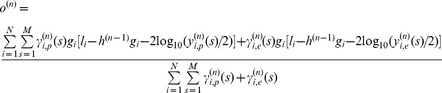
(7)This strategy works for four parameters appearing in emission probability function except the proportion of cancer cell *w*, as there is no close-form formula to update *w* in EM algorithm. However, this problem can be efficiently solved by using numerical methods such as Newton-Raphson method, which can numerically increase expectation of the partial log-likelihood in each M step.

### Significance Test for Tumor Aberrations

Given aberration profiles of M tumor samples, we need to find an efficient approach to evaluate the statistical significance of each aberration in cancer genome. For amplified aberrations, we used statistic 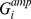 to reflect the amplitude and frequency of an amplified region in these tumor samples, which is defined as follow:
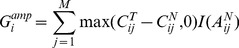
(8)



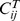
 and 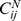 are 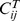
copy numbers at the *i^th^* probe in tumor sample *j* and matched normal sample, respectively. To investigate somatic amplifications, we require that this statistic only accounts for aberrations where there is no germline mutation in the corresponding genomic region of the matched normal sample. It is implemented by indicator function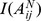, which returns 1 if state 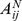 inferred by PSHMM is the normal state and 0 otherwise. Similarly, for deleted regions the statistic 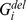 is formulated as follow:
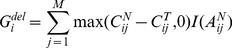
(9)


On the other hand, the statistic for LOH regions 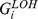 is defined as:
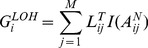
(10)where 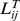 is the inferred LOH state in tumor sample.

To get the empirical distribution of statistic *G_i_* under the null hypothesis that the pinpointed region is not recurrent but random mutation, we adopt the procedure introduced in [6] by which the exact estimation of the null distribution can be efficiently obtained by using convolution of the statistic in tumor samples. Statistical significance of each aberration can then be evaluated by measuring the associated FDR q-value with a previously proposed threshold of 0.25 [6].

### Availability

An implementation of the proposed GIANT tool including PSHMM and statistic significance test of genomic aberrations using MATLAB/C is freely available from the associated website, together with supporting information (http://bioinformatics.ustc.edu.cn/giant/).

## Results

### Analysis of Replicate Tumor SNP Arrays

**Figure 1:**
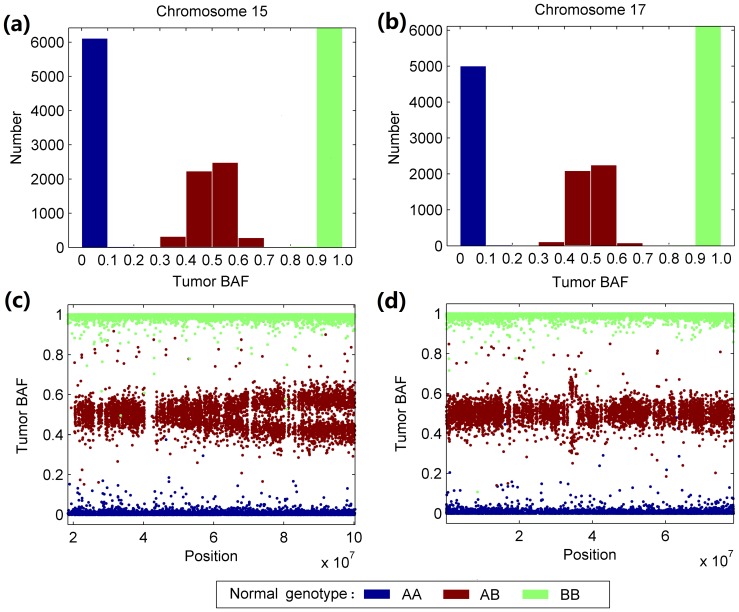
Statistical distributions of BAF signals in tumor replicate 1 with respect to corresponding normal genotypes. Genotypes are inferred by GIANT on paired replicate SNP array, and BAF signals, associated with corresponding normal genotypes, are illustrated. (a) Histogram of BAF signals for chromosome 15 with respect to different normal genotypes: ‘AA’ (blue), ‘AB’ (brown) and ‘BB’ (green). (b) Histogram of BAF signals for chromosome 17. (c) Spatial distribution of BAF signals for chromosome 15. (d) Spatial distribution of BAF signals for chromosome 17.

For comprehensive evaluation of GIANT’s robustness to genotyping signal variation and self-consistency in inference of normal genotypes and identification of genomic aberrations, we generated a replicate SNP array dataset representing one breast cancer and its matched normal sample. As the first step, we used the normal replicate SNP arrays to determine whether the genotypes inferred by GIANT are consistent. Figure S1 in File S1 shows that GIANT demonstrates good robustness to replicate arrays by providing very consistent results (>99.9% of total SNP probes). Moreover, we investigated the statistical distributions of BAF signals in tumor replicate 1 with respect to different normal genotypes (Figure 1). Clearly, the tumor genotyping profile follows the pattern of corresponding normal genotypes (‘AA’, ‘AB’ and ‘BB’). These results corroborate the intrinsic relationship between the genotypes of tumor and matched normal sample (i.e., normal genotype ‘AB’ can become any genotype in tumor, while genotype ‘AA’ (‘BB’) could generate only ‘A’, ‘AA’, ‘AAA’ etc. (‘B’, ‘BB’, ‘BBB’, etc.)). It should be pointed out that such information couldn’t be directly used to determine tumor genotypes and corresponding genomic aberrations. However, the normal genotype information can be incorporated into the emission probabilities of the PSHMM (Equation (1) and (3)) and therefore is helpful in modelling the genotyping signals of tumor sample and identifying genomic aberrations.

Furthermore, we applied GIANT to paired replicate SNP arrays. We examined the estimated parameters, as shown in Table S2 in File S1, and GIANT produces consistent estimation of parameters for all four replicate pairs. For example, as the key parameters for tumor SNP data analysis [21], [25]–[27], both the estimated cancer cell proportion *w* and LRR baseline shift *o* are concordant thorough all the tests. Besides, good self-consistency is observed in identifying genomic aberrations. For genomic aberrations on the q arm of chromosome 15, consistent results are also produced among different replicate pairs by GIANT (Figure S2 in File S1).

### Performance Evaluation using Simulated Data

First, we performed performance evaluation by applying GIANT to a widely-adopted simulated data set, which is originated from a diploid HapMap sample including ten synthetic aberrant regions differing in length, aberration type, etc [37]. Two “state of the art” methods for paired SNP array analysis: ASCAT [25] and OncoSNP [26], were also examined for comparison. Similar with GIANT, both methods are featured with automatic calibration for normal cell contamination and tumor aneuploidy. Normal cell contamination levels estimated by three investigated methods were compared to the underground truth, as illustrated in in Figure S3a and Table S3 in File S1. For a wide range of contamination level from 0% to 80%, the results of ASCAT show good concordance with the real values. However, no feasible solutions could be found by ASCAT for tumor samples with higher contamination level. On the other hand, results of OncoSNP are less consistent with the real contamination values, especially when there are more than 65% normal cells admixed in tumor sample. Compared to existing methods, GIANT demonstrates better performance in detecting normal contamination levels with more concordant results across all samples (correlation coefficient = 0.9996). We further explored tumor average copy number (ACN) estimated from the results of different methods (Figure S3b in File S1). In the case of low or medium normal cell contamination, all methods can correctly identify tumor samples as diploid. However, OncoSNP wrongly treats highly contaminated (greater than 65%) tumor samples as approximate triploid or even tetraploid, in concert with inaccurate estimation of normal cell contamination level. Among all tested methods, GIANT achieves the best consistency with contamination level up to 95%. More details of the results are provided in Table S3 in File S1.

**Figure 2:**
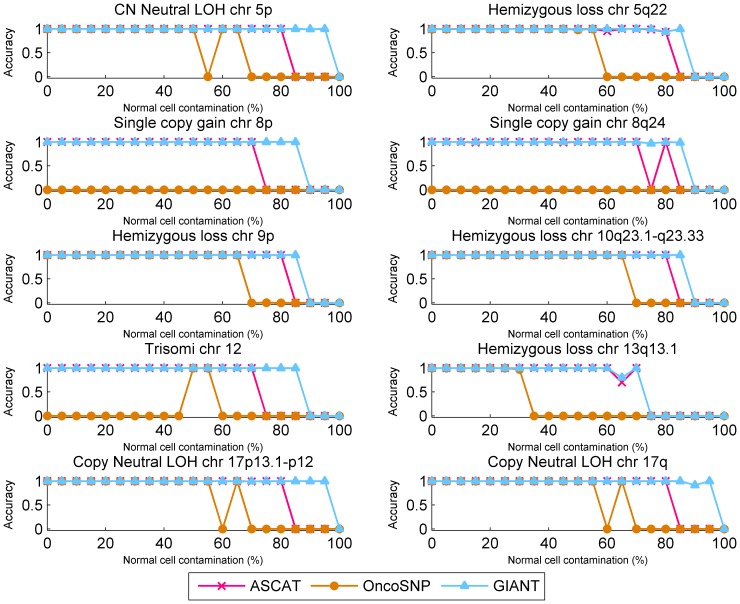
Comparison of accuracy for correctly identifying different types of aberration using simulated tumor SNP arrays. Accuracy is calculated by using the simulated tumor SNP arrays, which contains ten known altered regions with various aberration types, and results are illustrated for comparing the performance in identifying non-aberration regions with respect to increasing normal cell contamination levels. Different lines correspond to the accuracies for GIANT (blue), ASCAT (red) and OncoSNP (yellow).

Furthermore, we sought to illustrate how efficiently different methods distinguish aberrations from normal regions. The specificities of all investigated methods were calculated and plotted in Figure S4 in File S1, which are defined as the proportion of SNPs outside aberrant regions that are correctly identified as normal [33], [34], [37]. Overall, GIANT exhibits very high specificities (>0.985) throughout all tumor samples. Due to the systematic bias in tumor ACN determination, the specificity of OncoSNP declines sharply in highly contaminated tumor samples. ASCAT shows comparable performance to GIANT till normal cell contamination level reaches to 85%, after which acceptable solution is unavailable. Moreover, since all methods examined here can identify specific CNA/LOH types, we further calculated their accuracies defined as the proportion of correctly detected aberrant types for simulated regions, and the accuracies of ten aberrant regions were calculated and shown in Figure 2. GIANT consistently outperforms the other two methods throughout all tests. Especially, when normal cell contamination level reaches up to 95%, it can still correctly detect LOH regions with extremely high accuracy.

In addition, we made further comparison by recruiting two additional methods: PSCN [33] and PSCBS [34], which have been reported to have superior performance on this data set. Given the fact that they do not provide detection for different CNA/LOH types, in this case we used sensitivity as the performance measurement, which represents the proportion of aberrant regions correctly detected without taking into account aberration types. The results of all five approaches examined were illustrated in Figure S5 in File S1. Note that results with low specificities are not considered here as they render misleading sensitivities with incorrect aberration types. Again, GIANT achieves the best overall sensitivity especially in tumor samples with contamination level greater than 85%. Taken together, GIANT demonstrates a prominent advantage in the ability to accurately recognize diploid tumor samples and identify different types of aberrations, especially at high normal cell contamination level.

### Performance Evaluation Using Dilution Series of Cancer Cell-line

Next, to examine the performance of GIANT on real SNP array data, we adopted an experimental dilution series of CRL-2324 breast cancer cell line with matched normal cell line CRL-2325. It is of note that the cancer cell is highly aneuploid [37], therefore making data analysis a challenging task. In this regard, analysis of diluted aneuploid cancer cell-line will give rise to a more comprehensive understanding of the real potential of different methods investigated in this study. One intriguing phenomenon on the dilution series data is that broad hemizygous deletions spanning the whole 6p and 16q arms are reported in the matched normal cell line [26], [27], which further complicate genetic make-up of the mixed samples and thereby determination of aberrations in cancer cells. On the other hand, it also provides a good opportunity to investigate how tumor genotyping signal are affected by germline vairants in paired normal sample. Hence we first analyzed the statistical distributions of LRR and BAF signals in dilution series. From the genomic profiles of cancer and normal samples, we selected 12 manually annotated regions from 4 chromosomes including 6p and 16q, which are classified by four different scenarios: amplification/deletion, amplification/normal, deletion/normal, normal/normal. More detailed information of these regions is presented in Table S4 in File S1. We plotted real values of mean LRR/BAF for each region, as shown in Figure S6 in File S1. For comparison, we also calculated the theoretical mean values based on Equation (1) and (3) using parameters estimated by GIANT. The theoretical LRR/BAF means are favorably in concordance with the real values throughout a wide dilution range from 10% to 100%, suggesting the efficiency of the empirical formulas adopted in our statistical model.

Next, we applied GIANT, ASCAT and OncoSNP to dilution series data to evaluate whether they can correctly identify genome-wide aberrations from attenuated genotyping signals even as cancer cells diminish in the diluted samples. Since cancer cell contents in the dilution series are determined, they are used here for performance assessment. As shown in Table 1, the cancer cell contents recovered by GIANT are consistent with the real values throughout all dilution samples, even only 10% of cancer cells. Although ASCAT shows good performance when there are plenty of cancer cells admixed, it cannot find feasible solutions for three dilution samples with 21%, 14% and 10% cancer cells. Compared to GIANT, OncoSNP is less robust and sometimes generates apparently wrong results. On the other hand, all three methods correctly identify the cancer cell-line as aneuploid, but GIANT demonstrates clearly better self-consistency than other methods in ACN inference as well as identified LOH regions (Table 1). Finally, we examined our previously proposed GPHMM method [27], an efficient approach for single tumor SNP array analysis. We found GPHMM largely failed in this test with a total of 6 samples (cancer cell content<45%) erroneously identified. Given that GPHMM performs very well on the same data set with chromosome 6 and 16 removed [27], we conclude that the discrepancy between the performance of GIANT and GPHMM is caused by the variants on these two chromosomes that are not modeled in GPHMM. On the contrary, GIANT successfully addresses this issue by efficiently utilizing genotype information of the matched normal cell line.

**Table 1:** Summarized results of different methods for dilution samples.

Real cancer# (%)	GIANT			OncoSNP			ASCAT			GPHMM		
	Cancer$ (%)	ACN	LOH& (%)	Cancer$ (%)	ACN	LOH& (%)	Cancer$ (%)	ACN	LOH& (%)	Cancer$ (%)	ACN	LOH& (%)
10	9	2.72	71	20	3.34	1	n/a	n/a	n/a	85	1.95	5
14	11	2.66	62	20	3.19	27	n/a	n/a	n/a	77	1.96	5
21	17	2.92	58	30	3.09	43	n/a	n/a	n/a	39	3.55	5
23	30	2.88	57	40	2.94	61	28	2.82	63	23	3.71	62
30	26	2.89	58	40	2.90	57	25	2.87	61	60	3.58	1
34	31	2.86	58	40	3.03	58	29	2.85	64	72	3.57	1
45	35	2.86	59	50	2.91	59	34	2.80	63	35	2.76	61
47	43	2.85	58	60	2.92	60	43	2.81	64	44	2.80	59
50	41	2.85	58	50	3.21	58	41	2.77	64	42	2.78	59
79	77	2.85	58	80	3.22	57	77	2.79	58	78	2.82	58
100	97	2.81	58	100	3.11	58	98	2.95	60	93	2.88	58

# Real cancer = Real cancer cell content;

$ Cancer = Estimated cancer cell content;

& LOH = Estimated LOH proportion.

**Figure 3:**
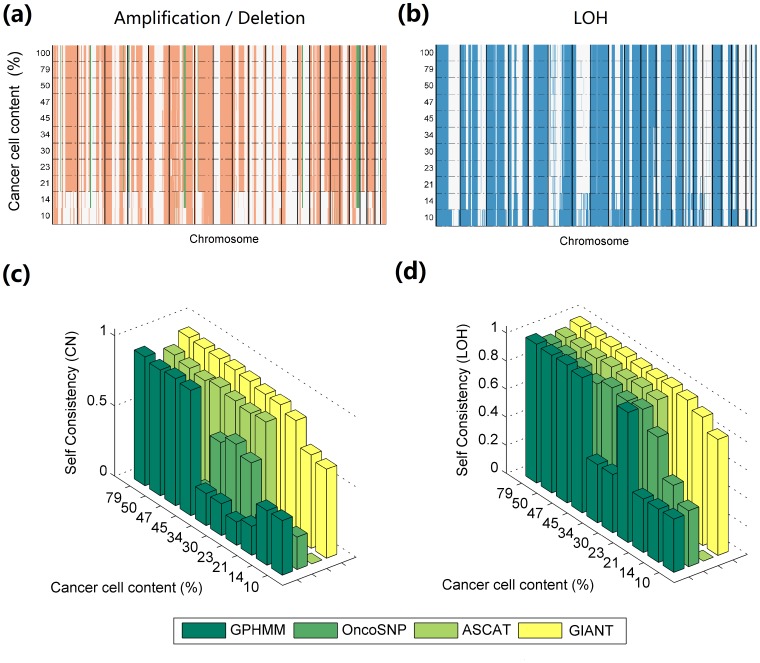
Assessment of robustness for different methods using dilution series data. Dilution series data of cancer cell line CRL-2324 and normal cell line CRL2325 are analyzed by GIANT for identifying genomic aberrations. (a) Genome-wide amplification (red)/deletion (green) profiles for dilution samples with cancer cell content ranging from 10% to 100%. (b) Genome-wide LOH (blue) profiles for dilution samples. The vertical stripes across different dilution levels reflect the strong reproducibility for aberration identification. (c) Self-consistency for copy number identification by GIANT (yellow), ASCAT (light green), OncoSNP (green) and GPHMM (dark green). (d) Self-consistency for LOH identification by GIANT ASCAT, OncoSNP and GPHMM.

To further assess the robustness of different methods, we inspected the whole-genome aberration profiles for all dilution samples, as shown in Figure 3a and 3b. GIANT demonstrates strong reproducibility, only with relatively more difference as cancer cell content declines to 14% and lower. A detailed analysis of these genomic profiles was performed by checking the aberrant regions on chromosome 6 and 13, which show very good agreement with known aberrant regions in CRL-2324 cell line, such as amplification on 13q13.1-mid q13, hemizygous loss on 6q14.3-mid q15 and 6q15-16, copy neutral LOH on 13q 14.2-13q14 and 13q21.31-qter [37] (see Figure S7 and S8 in File S1). Additionally, we measured the self-consistency of all four methods by paralleling the procedures previously reported [21], [26], [27], in which the results generated from diluted samples were compared to the results of pure cancer sample to determine the fraction of SNPs retaining the same copy number or LOH state. Figure 3c and 3d show that GIANT retains the highest self-consistency in identifying genomic aberrations across all dilution samples. This conclusion is also corroborated by the results of different methods for chromosome 1p (Figure S9 in File S1). The CNA and LOH identified by GIANT precisely reflect the complicated pattern of genotyping signals and are very consistent in different dilution samples, showing least discrepancy among all tested methods. In addition, we compare the self-consistency between GPHMM and GIANT by using the dilutions after removing the chromosome 6 and 16. Self-consistencies are calculated based on copy number state, LOH state, as shown in Figure S10 in File S1. We find that GIANT and GPHMM produce comparative results for the samples with high cancer cell proportion. When the proportion further decreases, such as 14% and 10%, GIANT demonstrates much higher self-consistencies than GPHMM for copy number identification. This result implies that the paired normal genotype information can be helpful for assisting the genomic aberration identification, especially when the cancer cell proportion is low.

**Figure 4:**
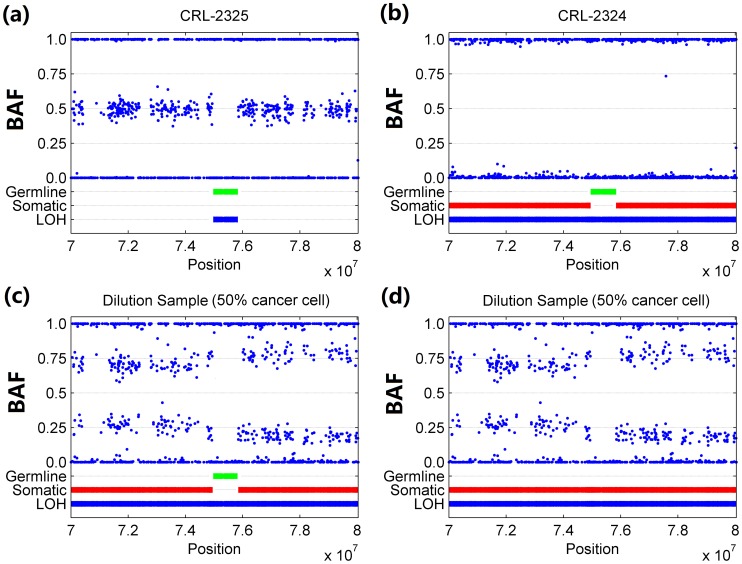
Analysis of germline/somatic aberrations on chromosome 14q24 of breast cancer cell line CRL-2324. Breast cancer cell line CRL-2324 and matched normal cell line CRL-2325 are analyzed by GIANT, and results on chromosome 14q24 are provided for demonstrating the performance in identifying the germline/somatic aberration. (a) Results of matched normal cell line CRL-2325 showing germline (green) LOH on this region. (b) Results of breast cancer cell line CRL-2324 showing this germline LOH is flanked by somatic (red) LOH. (c) Consistent results observed in dilution sample with 50% cancer cells. (d) Results of OncoSNP for the same dilution sample.

Beyond identifying aberrations in cancer genome, GIANT is capable of determining somatic/germline aberrations by checking whether aberrations detected in tumor sample also arise in matched normal sample. By using GIANT, we discovered a broad (>10Mb) somatic LOH region on 14q24 in CRL-2324 cell line that harbors a smaller (∼1 Mb) germline LOH region, as shown in Figure 4a and 4b. Note that in this case it would have been impossible to distinguish this germline aberration from flanking somatic aberration without genotype information of the matched normal sample. Moreover, the results of GIANT exhibit very good consistency in different dilution samples, as the example shown in Figure 4c. In contrast, Figure 4d shows OncoSNP wrongly identifies the whole LOH region as somatic. Based on the comprehensive analysis discussed above, we come to the conclusion that for aneuploid samples, GIANT demonstrates robustness to tumor aneuploidy and normal cell contamination, and superior performance in identifying different kinds of genomic aberrations.

### Analysis of Individual Tumor Samples

**Figure 5:**
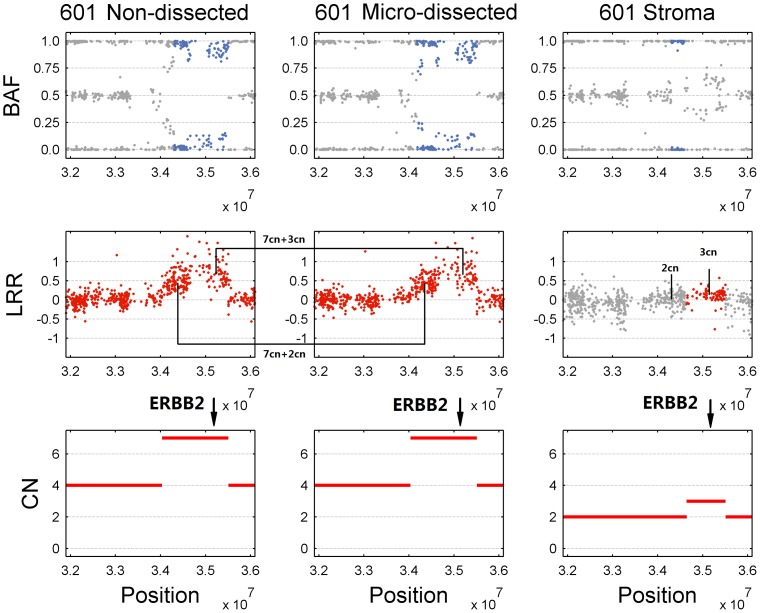
Identification of genomic aberrations in ERBB2 gene for breast tumor 601. Both micro-dissected and non-dissected tumor samples of HER2-positive breast cancer 601 are analyzed by GIANT with paired stroma sample. LRR/BAF signals and GIANT results around gene ERBB2 are provided for non-dissected tumor sample (left panel), dissected tumor sample (middle panel) and stroma sample (right panel).

GIANT was examined by a number of individual tumor samples and the results are shown to be helpful for interpretation of tumor SNP array data. For example, we analyzed the breast tumor in replicate SNP array dataset and the tumor content detected by GIANT was only 10% in this sample, which was concordant with the result of manual annotation suggesting it was highly contaminated by normal cells. Note in this case, the genotyping signals for cancer cells are extremely attenuated and therefore detection of genomic aberration becomes very difficult and at the same time prone to noise in SNP array data. However, GIANT successfully identified amplification on chromosome 17 that harbored ERBB2 (HER2) oncogene (see Figure S11 in File S1), indicating this breast tumor was HER2-positive. Furthermore, the results of GIANT also showed that it was actually a somatic aberration, as the corresponding region in matched normal sample remained unchanged. On the contrary, when another HER2-positive breast tumor (case 601) described in [26] was tested, with the help of GIANT we found that HER2 was amplified not only in tumor but also in normal stroma (Figure 5), indicating that the stroma sample may actually be contaminated by the tumor cells due to imperfect microdissection.

**Figure 6:**
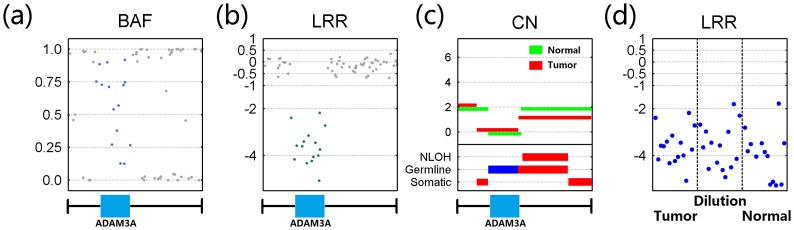
Identification of homozygous deletion in ADAM3A gene for tumor 7207. Breast tumor sample 7207, as well as the dilution sample, is analyzed by GIANT with match normal sample, and results around gene ADAM3A are provided for demonstrating the performance in identifying the germline aberration. (a) Altered BAF signal in tumor sample. (b) Altered LRR signal in tumor sample. (c) Visualized results of GIANT showing germline homozygous deletion with zero copy of ADAM3A in both normal (green) and tumor (red) sample. (d) Similar LRR patterns for this region in tumor, normal and dilution samples.

Another sample showing the utility of GIANT is the study of tumor sample 7207 as described in [19]. We conducted an analysis of ADAM3A aberration in this tumor and paired normal sample by using GIANT. The results from the GIANT revealed that this area was consistently identified as the deletion in both normal and tumor samples (As shown in Figure 6 abc), implying the possibility of occurrence of germline aberration, and this was further verified by a parallel analysis of a diluted sample of the same tumor (see Figure S12 in File S1). As shown in Figure 6d, germline homozygous deletion leads to similar patterns of LRR signal for this region in tumor, normal and dilution samples. For comparison, we also applied ASCAT and OncoSNP on this data and the results of chromosome 8 are plotted in Figure S13 in File S1. ASCAT is capable of detecting the homozygous deletion, but wrongly classifies breast tumor 7207 as tetraploid with a large proportion (>60%) of cancer cells. In contrast, OncoSNP correctly identifies tumor ACN and cancer cell content, but fails to recognize the homozygous deletion of ADAM3A.

### Application of GIANT to Urothelial Cancer Samples

**Figure 7:**
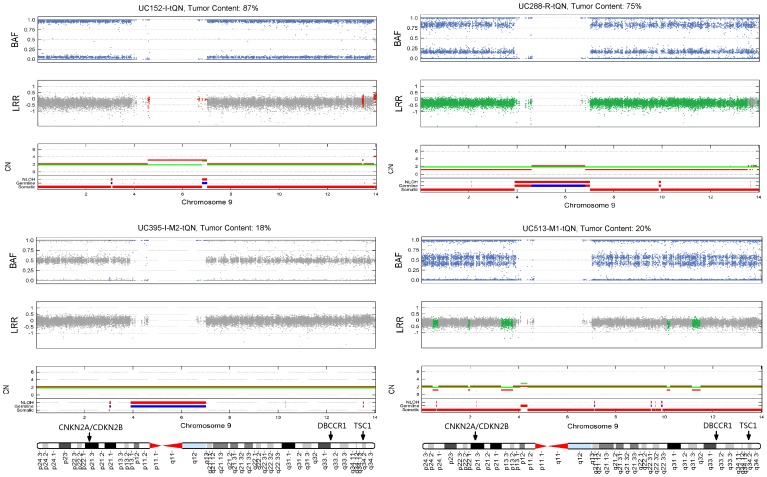
Genomic copy number and LOH profiles for chromosome 9 of four urothelial tumors. The results of GIANT for chromosome 9 in four urothelial tumors are illustrated. Broad LOH regions (colored as blue in BAF) identified by GIANT are found in three tumor samples. Numerous CNA regions (colored in LRR, green for deletion, red for amplification) are also identified in tumor samples. The CN panel shows copy number of cancer (red) and matched normal (green) sample. Additional information, such as LOH status in normal sample, germline aberration status (blue for consistent aberration types in both normal and tumor sample, red for inconsistent aberration types) and somatic aberration status (denoting the region where aberration only occurs in tumor sample), are provided at the bottom of CN panel.

In practice, analysis of genomic aberrations is often performed on multiple tumors at a time. As such, we undertook an investigation on a data set including four urothelial tumors with matched normal sample [37]. Figure 7 shows visualized results retrieved by GIANT for a comparative inspection, and in three tumor samples there are broad LOH spanning the whole p and q arm of chromosome 9. In contrast, recurrent somatic CNAs were not observed in the whole chromosome. By checking the Atlas of Genetics and Cytogenetics in Oncology and Haematology [8], we found numerous LOH regions on chromosome 9 were reported in urothelial cancer. For example, 9p21 and 9q32-34 are two well-known LOH regions that harbor genes implicated in urothelial cancer such as CDKN2A/CDKN2B, TSC1, DBCCR1 (Figure 7). More detailed results of identified somatic aberrations for a list of known aberrant regions are presented in Table S5 in File S1.

Although useful in this case, visual inspection is not always easily applicable to studies especially with a large number of tumor samples. Hence we performed a genome-wide permutation test and the statistical significance of somatic aberrations was then evaluated by q-values with a pre-defined threshold of 0.25 [6]. Three previously reported LOH regions in urothelial cancer, 9p21, 9q32-34 and 4p16.3, were significant in these tumors (see Figure S14 in File S1). Other significant LOH regions on chromosome 9, such as 9p24-23 and 9p13, also indicate possible association with urothelial cancer. For CNA regions, there are no significantly amplified regions detected, probably due to limited sample size. On the other hand, significance test of somatic deletion in urothelial cancer led to two new candidate regions: 6p21.3 and 8p21 (illustrated in Figure S15 in File S1), but after further examination it became clear that deletion on 6p21.3 was indeed spurious aberration that was caused by severe genotyping errors in tumor samples. Whilst homozygous deletion on 8p21 was observed in two tumor samples, indicating possible dysfunction of cancer-related genes on this region.

### Application of GIANT to Breast Cancer Samples

**Figure 8:**
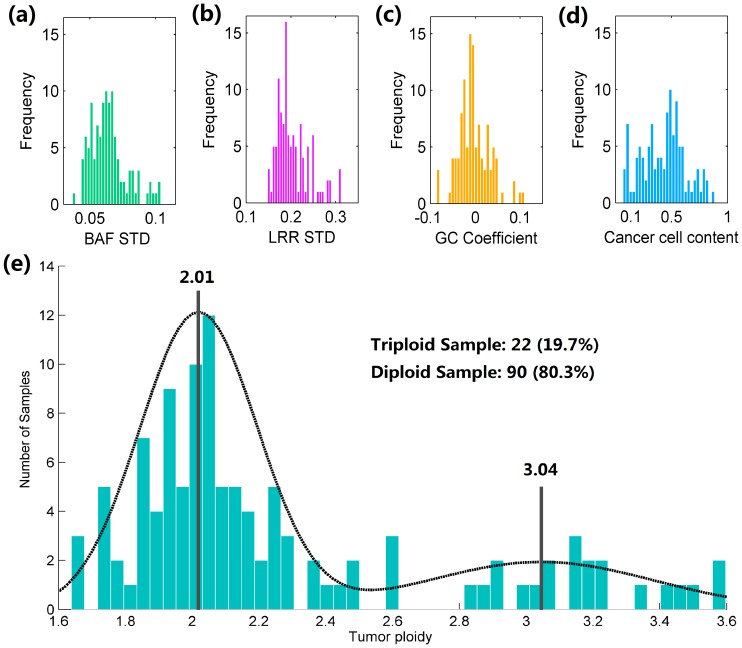
Histograms of summarized results for 112 breast cancer samples. Histograms of summarized results provided by GIANT, includes (a) standard deviation of BAF, (b) standard deviation of LRR, (c) coefficient for local GC content, (d) proportion of cancer cells admixed in tumor sample and (e) tumor ACN for 112 breast cancer samples. Dashed line shows the fitted Gaussian mixture models (Group I and II). Solid lines represent mean average copy number of group I and II respectively.

We further applied GIANT to a large SNP array data set consisting of 112 breast tumors matched by peripheral blood [25]. The histograms of summarized results are plotted in Figure 8. Although the distributions of standard deviation (STD) of LRR and BAF (Figure 8a and 8b) indicate generally good signal quality of these tumor samples, the GC coefficients shown in Figure 8c suggest that signal noise associated with local GC bias in some tumor samples are not trivial. In addition, we found that about 9% of tumor samples (10 of 112) had less than 10% of cancer cells (Figure 8d). On the other hand, it was reported that ASCAT could not find feasible solutions for 21 tumor samples [25] and this number dropped to 17 when the current version (2.1) was used. We further compared these 17 tumor samples to the remaining samples using the results of GIANT, and discovered that there was actually no significant difference in signal STD (t-test, LRR p-value: 0.13, BAF p-value: 0.74, see Figure S16 in File S1). However, the median of tumor content for unresolved samples is 16%, which is significantly lower than that of the remaining tumor samples with feasible solution (t-test, p-value: 1.3×10–9, Figure S16 in File S1). These results suggest that infeasible solutions are caused by severe normal cell contamination, and similar conclusion is also made in another study of tumor cellularity [29].

Next, we assessed the distribution of estimated tumor ACN from the results of GIANT. Similar to the findings in [38] and [16], Figure 8e shows that tumor ACN is apparently not normally distributed but has two distinct peaks. Fitting by Gaussian mixture model (see dashed line representing the fitted model) gives rise to two normal distributions with mean ACN of 2.01 and 3.04, suggesting these tumor samples can be categorized into two groups (hereafter called Group I and Group II), with the respective sample number being 90 and 22. Moreover, most of the tumors in Group I exhibit a so-called “simplex” CNA pattern [5] (depicted in Figure S17 in File S1) characterized by broad amplifications and deletions up to the whole chromosome arms, whereas many genomic profiles of the tumors in Group II show a discrepant “sawtooth” pattern [5] featured by a largely aberrant genome with many narrow amplifications and deletions (depicted in Figure S17 in File S1). The genomic profiles of all breast tumors illustrated in Figure S18 in File S1 show that the tumors in Group II demonstrate genome-wide amplifications while the tumors in Group I in general have less amplifications but more deletions. In addition, distinct patterns of aberrations on particular chromosome regions are also observed. For example, on the q arms of chromosome 1 and 8 broad amplifications are dominant in both two groups of tumors. While on the q arms of chromosome 11 and 16, amplifications are only observed in Group II tumors but deletions are abundant in Group I tumors.

**Figure 9:**
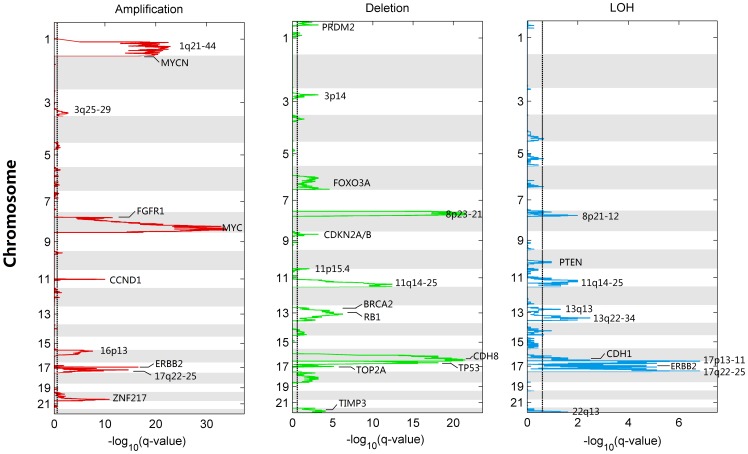
Genome-wide analysis of statistically significant somatic aberrations for breast cancer samples. 112 breast tumor samples are assessed by GIANT for identifying the significant aberrations. Statistical significance for amplification (left panel), deletion (middle panel) and LOH (right panel) was evaluated by using FDR corrected q-value. Dash lines correspond to the significant threshold of 0.25.

Following the approaches for investigating genomic aberrations [5], [7], [10]–[12], [25], we plotted the frequencies of somatic amplification, deletion and LOH of all breast cancer samples, as illustrated in Figure S19 and S20 in File S1. Consistent with the findings in previous studies [5], [7], [25], we observed frequent amplification (1q, 8q, 16p), deletion (8p, 11q, 16q and 17p) and LOH (8p, 11q, 16q and 17p) in breast cancer. However, these results directly obtained from eyeballing of frequency plots are very coarse. More problematically, relatively high frequency does not always lead to statistical significance and vice versa. Therefore we further performed statistical significance analysis of genome-wide somatic aberrations. As shown in Figure 9, the q-values of CNA calculated by GIANT demonstrate numerous significantly altered regions, which harbor many breast cancer associated oncogenes and tumor suppressor genes such as ERBB2, MYC, CCND1, BRCA2, TP53, PTEN [5], [7], [8]. Note that although chromosome 3q, 11q and 14q all contain aberrant regions associated with high amplification frequencies (35–40%, see Figure S19 in File S1), the statistical test suggests only the region on 11q that corresponds to oncogene CCND1 is indeed significantly amplified. In addition, the results of statistical analysis show both amplification and LOH on 17q12 are significant, indicating only one of the two alleles for ERBB2 oncogene tends to be exclusively duplicated in these breast tumors. This mono-allelic amplification phenomenon is also observed in another study on ERBB2-amplified tumors [9]. Finally, a full list of significant aberrations identified by GIANT accompanied with more detailed information is provided in Table S6 in File S1.

## Discussion

We present an efficient computational method for analyzing paired SNP array data generated from tumor and matched normal samples. By incorporating genotype information from normal SNP array data, GIANT can accurately identify genome-wide somatic aberrations in cancer genome, even for highly contaminated aneuploid tumor samples. In various tests conducted in this study, the performance of GIANT is superior to existing methods. In addition, GIANT provides statistical significance analysis of tumor aberrations for systematic discovery of recurrent mutations.

GIANT takes full advantage of the underlying genetic relationship of tumor and matched normal sample, and at the same time address critical issues including tumor aneuploidy and normal cell contamination simultaneously. Our results demonstrate that genotype information obtained from normal sample is helpful in determining tumor genotypes and corresponding aberrations, thereby rendering more accurate models with better parameter estimation and improved performance. We would like to point out that the strategy of utilizing genotypes of matched normal sample for tumor aberration detection does not exclude other computational approaches that aims to remove noise in tumor genotyping signals by using normal SNP array data, e.g., the noise template adopted in OncoSNP. Therefore, if necessary they may serve as a pre-processing procedure to improve the quality of genotyping signals prior to the application of GIANT. On the other hand, it is of note that in practice GIANT shows reasonable robustness to heterogeneous tumor samples, e.g., the dilution series (tumor heterogeneity observed in q arm of chromosome 1), but the statistical model in GIANT does not explicitly account for tumor heterogeneity and therefore multiple cancer subclones in tumor sample may in fact lead to erroneous parameter estimation and inaccurate detection of genomic aberrations. Although in principle it is possible to address the issue of tumor heterogeneity, for example, by adopting the intra-tumor heterogeneity model [26] used in OncoSNP, it should be stressed that extensive modelling of tumor heterogeneity will inevitably bring more variations, e.g., specific proportions and genomic aberrations associated with different cancer subclones, into the whole framework and therefore may result in over-fitting problems. Hence more sophisticated methods are required in tumor SNP array data analysis when tumor heterogeneity is taken into consideration.

In this study, the tumor samples were analyzed by Illumina SNP arrays, but GIANT is not restricted to Illumina platform. Similar with other tools such as ASCAT and OncoSNP, GIANT can be generally applied to handle SNP array data generated by Affymetrix platform with the help of specific conversion tools such as PennCNV [39] and AROMA CRMAv2 [40]. Also the statistical significance test adopted in GIANT is platform independent and therefore can be easily used to find recurrent aberrations from the results of Affymetrix SNP arrays. At the same time, the work described in this paper can also inspire research on computational tools for tumor NGS data analysis, which has been greatly benefited from the studies of tumor SNP arrays [16], [17]. So far, some aberration detection methods initially designed for SNP arrays, such as GAP [21] and TAPS [28], have been successfully transferred to NGS platform [15], [18]. Our next step in the future is to adopt the statistical framework of GIANT in NGS data analysis, and given many advantageous factors, such as the similarity of concept and methodology for read-depth (density of short reads aligned along the genome) and microarray based approaches in identifying genomic aberrations [15], [18], availability of BAF from NGS data [15] and platform independency of statistical significance analysis, we believe this approach will be highly feasible.

In conclusion, GIANT represents a valuable and powerful bioinformatics resource for identifying important aberrations in cancer genome, which may help to elucidate critical events contributing to tumorigenesis and at the same time show its usefulness in personalized targeted therapy for cancer.

## Supporting Information

File S1 **File S1 contains the following files. Text S1. Detailed description of statistical methods in GIANT. Figure S1. Comparison of BAF signals in two normal replicates on different chromosomes. Figure S2. Visualized genomic aberrations for replicate pairs. Figure S3. Comparison of estimated normal cell contamination levels and tumor average copy number for simulated tumor SNP arrays. Figure S4. Comparison of specificity for identifying normal regions in simulated tumor samples. Figure S5 Sensitivities for distinguishing aberrations from normal regions by five different methods. Figure S6. Theoretical and real mean values of LRR/BAF in different scenarios. Figure S7. Aberrations in chromosome 6 of breast cancer cell line CRL-2324. Figure S8. Aberrations in chromosome 13 of breast cancer cell line CRL-2324. Figure S9. Comparison of results for breast cancer cell line CRL-2324 by different methods. Figure S10. Comparison of self-consistency for GIANT and GPHMM using dilution series data. Figure S11. Visualized results of GIANT for replicate breast cancer sample. Figure S12. Detection of germline homozygous deletion of ADAM3A gene in diluted breast tumor sample 7207. Figure S13. Comparison of the results for chromosome 8 of breast tumor 7207 by different methods. Figure S14. Significance test of genome-wide somatic amplification and LOH for urothelial cancer samples. Figure S15. Significance test of genome-wide somatic deletion for urothelial cancer samples. Figure S16. Box plots of summarized results for 112 breast cancer samples. Figure S17. Distinct patterns of genome-wide copy number profiles identified in two groups of tumors. Figure S18. Genome-wide CNA profiles of breast cancer samples. Figure S19. Frequency plot of genomic-wide CNA for 112 breast cancer samples. Figure S20. Frequency plot of genomic-wide LOH for 112 breast cancer samples. Table S1. Detailed information of hidden states in PSHMM. Table S2. Parameters estimated by GIANT on replicate SNP arrays. Table S3. Details of estimated parameters from paired methods. Table S4. Genomic regions used for illustrating real distributions of LRR/BAF mean values.** This file contains a supplementary table with detailed information of 12 genomic regions, including chromosome number, start and end position, aberration type, copy number and mean values of LRR/BAF signal in dilution series. **Table S5. Genomic aberrations in known abnormal regions implicated in urothelial cancer. Table S6. Detailed results for significance test of genome-wide aberration in 112 breast cancer samples.** This table contains detailed information of significant aberrant regions identified by GIANT.
(ZIP)

## References

[1] Stratton MR, Campbell PJ, Futreal PA (2009) The cancer genome. Nature 458: 719–724. 19360079 10.1038/nature07943 PMC2821689

[2] Albertson DG, Collins C, McCormick F, Gray JW (2003) Chromosome aberrations in solid tumors. Nat Genet 34: 369–376. 12923544 10.1038/ng1215

[3] Bignell GR, Greenman CD, Davies H, Butler AP, Edkins S, et al (2010) Signatures of mutation and selection in the cancer genome. Nature 463: 893–898. 20164919 10.1038/nature08768 PMC3145113

[4] Stephens PJ, McBride DJ, Lin ML, Varela I, Pleasance ED, et al (2009) Complex landscapes of somatic rearrangement in human breast cancer genomes. Nature 462: 1005–1010. 20033038 10.1038/nature08645 PMC3398135

[5] Hicks J, Krasnitz A, Lakshmi B, Navin NE, Riggs M, et al (2006) Novel patterns of genome rearrangement and their association with survival in breast cancer. Genome Res 16: 1465–1479. 17142309 10.1101/gr.5460106 PMC1665631

[6] Beroukhim R, Getz G, Nghiemphu L, Barretina J, Hsueh T, et al (2007) Assessing the significance of chromosomal aberrations in cancer: methodology and application to glioma. Proc Natl Acad Sci U S A 104: 20007–20012. 18077431 10.1073/pnas.0710052104 PMC2148413

[7] Haverty PM, Fridlyand J, Li L, Getz G, Beroukhim R, et al (2008) High-resolution genomic and expression analyses of copy number alterations in breast tumors. Genes Chromosomes Cancer 47: 530–542. 18335499 10.1002/gcc.20558

[8] Beroukhim R, Mermel CH, Porter D, Wei G, Raychaudhuri S, et al (2010) The landscape of somatic copy-number alteration across human cancers. Nature 463: 899–905. 20164920 10.1038/nature08822 PMC2826709

[9] Staaf J, Jonsson G, Ringner M, Vallon-Christersson J, Grabau D, et al (2010) High-resolution genomic and expression analyses of copy number alterations in HER2-amplified breast cancer. Breast Cancer Res 12: R25. 20459607 10.1186/bcr2568 PMC2917012

[10] Mermel CH, Schumacher SE, Hill B, Meyerson ML, Beroukhim R, et al (2011) GISTIC2.0 facilitates sensitive and confident localization of the targets of focal somatic copy-number alteration in human cancers. Genome Biol 12: R41. 21527027 10.1186/gb-2011-12-4-r41 PMC3218867

[11] Staaf J, Jonsson G, Ringner M, Baldetorp B, Borg A (2011) Landscape of somatic allelic imbalances and copy number alterations in HER2-amplified breast cancer. Breast Cancer Res 13: R129. 22169037 10.1186/bcr3075 PMC3326571

[12] Yuan X, Yu G, Hou X, Shih Ie M, Clarke R, et al (2012) Genome-wide identification of significant aberrations in cancer genome. BMC Genomics 13: 342. 22839576 10.1186/1471-2164-13-342 PMC3428679

[13] Park PJ (2008) Experimental design and data analysis for array comparative genomic hybridization. Cancer Invest 26: 923–928. 19034774 10.1080/07357900801993432

[14] Peiffer DA, Le JM, Steemers FJ, Chang W, Jenniges T, et al (2006) High-resolution genomic profiling of chromosomal aberrations using Infinium whole-genome genotyping. Genome Res 16: 1136–1148. 16899659 10.1101/gr.5402306 PMC1557768

[15] Boeva V, Popova T, Bleakley K, Chiche P, Cappo J, et al (2012) Control-FREEC: a tool for assessing copy number and allelic content using next-generation sequencing data. Bioinformatics 28: 423–425. 22155870 10.1093/bioinformatics/btr670 PMC3268243

[16] Carter SL, Cibulskis K, Helman E, McKenna A, Shen H, et al (2012) Absolute quantification of somatic DNA alterations in human cancer. Nat Biotechnol 30: 413–421. 22544022 10.1038/nbt.2203 PMC4383288

[17] Ha G, Roth A, Lai D, Bashashati A, Ding J, et al (2012) Integrative analysis of genome-wide loss of heterozygosity and monoallelic expression at nucleotide resolution reveals disrupted pathways in triple-negative breast cancer. Genome Res 22: 1995–2007. 22637570 10.1101/gr.137570.112 PMC3460194

[18] Mayrhofer M, Dilorenzo S, Isaksson A (2013) Patchwork: allele-specific copy number analysis of whole-genome sequenced tumor tissue. Genome Biol 14: R24. 23531354 10.1186/gb-2013-14-3-r24 PMC4053982

[19] Curtis C, Lynch AG, Dunning MJ, Spiteri I, Marioni JC, et al (2009) The pitfalls of platform comparison: DNA copy number array technologies assessed. BMC Genomics 10: 588. 19995423 10.1186/1471-2164-10-588 PMC2797821

[20] Nancarrow DJ, Handoko HY, Stark MS, Whiteman DC, Hayward NK (2007) SiDCoN: a tool to aid scoring of DNA copy number changes in SNP chip data. PLoS One 2: e1093. 17971856 10.1371/journal.pone.0001093 PMC2034603

[21] Popova T, Manie E, Stoppa-Lyonnet D, Rigaill G, Barillot E, et al (2009) Genome Alteration Print (GAP): a tool to visualize and mine complex cancer genomic profiles obtained by SNP arrays. Genome Biol 10: R128. 19903341 10.1186/gb-2009-10-11-r128 PMC2810663

[22] Sun W, Wright FA, Tang Z, Nordgard SH, Van Loo P, et al (2009) Integrated study of copy number states and genotype calls using high-density SNP arrays. Nucleic Acids Res 37: 5365–5377. 19581427 10.1093/nar/gkp493 PMC2935461

[23] Greenman CD, Bignell G, Butler A, Edkins S, Hinton J, et al (2010) PICNIC: an algorithm to predict absolute allelic copy number variation with microarray cancer data. Biostatistics 11: 164–175. 19837654 10.1093/biostatistics/kxp045 PMC2800165

[24] Liu Z, Li A, Schulz V, Chen M, Tuck D (2010) MixHMM: inferring copy number variation and allelic imbalance using SNP arrays and tumor samples mixed with stromal cells. PLoS One 5: e10909. 20532221 10.1371/journal.pone.0010909 PMC2879364

[25] Van Loo P, Nordgard SH, Lingjaerde OC, Russnes HG, Rye IH, et al (2010) Allele-specific copy number analysis of tumors. Proc Natl Acad Sci U S A 107: 16910–16915. 20837533 10.1073/pnas.1009843107 PMC2947907

[26] Yau C, Mouradov D, Jorissen RN, Colella S, Mirza G, et al (2010) A statistical approach for detecting genomic aberrations in heterogeneous tumor samples from single nucleotide polymorphism genotyping data. Genome Biol 11: R92. 20858232 10.1186/gb-2010-11-9-r92 PMC2965384

[27] Li A, Liu Z, Lezon-Geyda K, Sarkar S, Lannin D, et al (2011) GPHMM: an integrated hidden Markov model for identification of copy number alteration and loss of heterozygosity in complex tumor samples using whole genome SNP arrays. Nucleic Acids Res 39: 4928–4941. 21398628 10.1093/nar/gkr014 PMC3130254

[28] Rasmussen M, Sundstrom M, Goransson Kultima H, Botling J, Micke P, et al (2011) Allele-specific copy number analysis of tumor samples with aneuploidy and tumor heterogeneity. Genome Biol 12: R108. 22023820 10.1186/gb-2011-12-10-r108 PMC3333778

[29] Song S, Nones K, Miller D, Harliwong I, Kassahn KS, et al (2012) qpure: A tool to estimate tumor cellularity from genome-wide single-nucleotide polymorphism profiles. PLoS One 7: e45835. 23049875 10.1371/journal.pone.0045835 PMC3457972

[30] Diskin SJ, Li M, Hou C, Yang S, Glessner J, et al (2008) Adjustment of genomic waves in signal intensities from whole-genome SNP genotyping platforms. Nucleic Acids Res 36: e126. 18784189 10.1093/nar/gkn556 PMC2577347

[31] Attiyeh EF, Diskin SJ, Attiyeh MA, Mosse YP, Hou C, et al (2009) Genomic copy number determination in cancer cells from single nucleotide polymorphism microarrays based on quantitative genotyping corrected for aneuploidy. Genome Res 19: 276–283. 19141597 10.1101/gr.075671.107 PMC2652209

[32] Bengtsson H, Neuvial P, Speed TP (2010) TumorBoost: normalization of allele-specific tumor copy numbers from a single pair of tumor-normal genotyping microarrays. BMC Bioinformatics 11: 245. 20462408 10.1186/1471-2105-11-245 PMC2894037

[33] Chen H, Xing H, Zhang NR (2011) Estimation of parent specific DNA copy number in tumors using high-density genotyping arrays. PLoS Comput Biol 7: e1001060. 21298078 10.1371/journal.pcbi.1001060 PMC3029233

[34] Olshen AB, Bengtsson H, Neuvial P, Spellman PT, Olshen RA, et al (2011) Parent-specific copy number in paired tumor-normal studies using circular binary segmentation. Bioinformatics 27: 2038–2046. 21666266 10.1093/bioinformatics/btr329 PMC3137217

[35] Nilsen G, Liestol K, Van Loo P, Moen Vollan HK, Eide MB, et al (2012) Copynumber: Efficient algorithms for single- and multi-track copy number segmentation. BMC Genomics 13: 591. 23442169 10.1186/1471-2164-13-591 PMC3582591

[36] Ortiz-Estevez M, Aramburu A, Bengtsson H, Neuvial P, Rubio A (2012) CalMaTe: a method and software to improve allele-specific copy number of SNP arrays for downstream segmentation. Bioinformatics 28: 1793–1794. 22576175 10.1093/bioinformatics/bts248 PMC3381965

[37] Staaf J, Lindgren D, Vallon-Christersson J, Isaksson A, Goransson H, et al (2008) Segmentation-based detection of allelic imbalance and loss-of-heterozygosity in cancer cells using whole genome SNP arrays. Genome Biol 9: R136. 18796136 10.1186/gb-2008-9-9-r136 PMC2592714

[38] Popova T, Manie E, Rieunier G, Caux-Moncoutier V, Tirapo C, et al (2012) Ploidy and large-scale genomic instability consistently identify basal-like breast carcinomas with BRCA1/2 inactivation. Cancer Res 72: 5454–5462. 22933060 10.1158/0008-5472.CAN-12-1470

[39] Wang K, Li M, Hadley D, Liu R, Glessner J, et al (2007) PennCNV: an integrated hidden Markov model designed for high-resolution copy number variation detection in whole-genome SNP genotyping data. Genome Res 17: 1665–1674. 17921354 10.1101/gr.6861907 PMC2045149

[40] Bengtsson H, Wirapati P, Speed TP (2009) A single-array preprocessing method for estimating full-resolution raw copy numbers from all Affymetrix genotyping arrays including GenomeWideSNP 5 & 6. Bioinformatics 25: 2149–2156. 19535535 10.1093/bioinformatics/btp371 PMC2734319

