# Supplementary material for: Genome-Wide Identification of Somatic Aberrations from Paired Normal-Tumor Samples
Source: PLoS One. 2014 Jan 30;9(1):e87212. doi: 10.1371/journal.pone.0087212 (PMC3907544; doi:10.1371/journal.pone.0087212)
Supplement: File S1 — contains the following files. Text S1. Detailed description of statistical methods in GIANT. Figure S1. Comparison of BAF signals in two normal replicates on different chromosomes. Figure S2. Visualized genomic aberrations for replicate pairs. Figure S3. Comparison of estimated normal cell contamination levels and tumor average copy number for simulated tumor SNP arrays. Figure S4. Comparison of specificity for identifying normal regions in simulated tumor samples. Figure S5 Sensitivities for distinguishing aberrations from normal regions by five different methods. Figure S6. Theoretical and real mean values of LRR/BAF in different scenarios. Figure S7. Aberrations in chromosome 6 of breast cancer cell line CRL-2324. Figure S8. Aberrations in chromosome 13 of breast cancer cell line CRL-2324. Figure S9. Comparison of results for breast cancer cell line CRL-2324 by different methods. Figure S10. Comparison of self-consistency for GIANT and GPHMM using dilution series data. Figure S11. Visualized results of GIANT for replicate breast cancer sample. Figure S12. Detection of germline homozygous deletion of ADAM3A gene in diluted breast tumor sample 7207. Figure S13. Comparison of the results for chromosome 8 of breast tumor 7207 by different methods. Figure S14. Significance test of genome-wide somatic amplification and LOH for urothelial cancer samples. Figure S15. Significance test of genome-wide somatic deletion for urothelial cancer samples. Figure S16. Box plots of summarized results for 112 breast cancer samples. Figure S17. Distinct patterns of genome-wide copy number profiles identified in two groups of tumors. Figure S18. Genome-wide CNA profiles of breast cancer samples. Figure S19. Frequency plot of genomic-wide CNA for 112 breast cancer samples. Figure S20. Frequency plot of genomic-wide LOH for 112 breast cancer samples. Table S1. Detailed information of hidden states in PSHMM. Table S2. Parameters estimated by GIANT on replicate SNP arrays. Table S [file pone.0087212.s001.zip › TableS5.pdf]

Table S5: Genomic aberrations in known abnormal regions implicated in urothelial cancer.

This table contains a supplementary table of the detailed results of GIANT for some genomic regions implicated in urothelial cancer.

Region	Reported Aberration	UC152_I		UC288_R		UC395_I_M2		UC513_M1		Oncogene or suppressor gene
		Somatic Aberration	Type	Somatic Aberration	Type	Somatic Aberration	Type	Somatic Aberration	Type	
1p36	Amplification	Amplification	Broad	-	-	-	-	Amplification	Focal	TP73
4p16.3	LOH	Amplification, LOH	Broad	LOH	Broad	-	-	Amplification, LOH	Broad	FGFR3
6p22	Amplification	Amplification	Focal	-	-	-	-	Amplification	Broad	E2F3
8p12-22	Amplification	Amplification, LOH	Focal	-	-	-	-	Amplification	Broad	FGFR1, CMYC
9p21	Deletion LOH	LOH	Broad	Deletion	Broad	-	-	LOH	Broad	CDKN2A, CDKN2B
9q32-34	LOH	LOH	Broad	LOH	Broad	-	-	LOH	Broad	TSC1, DBCCR1
10p13	Amplification	Amplification	Broad	-	-	-	-	Amplification	Broad	-
10q23	Deletion	LOH	Broad	-	-	-	-	Amplification	Broad	PTEN
11q13	Amplification	Amplification	Broad	-	-	-	-	Amplification	Focal	CCND1, EMS1, INT2
13q14	Deletion	LOH	Broad	-	-	Deletion	Focal	Amplification	Broad	RB1
17p13	Deletion	LOH	Broad	Deletion	Broad	-	-	Amplification	Broad	TP53
17q21	Amplification	Amplification	Broad	-	-	-	-	Amplification	Broad	ERBB2
19q13.1	Amplification	Amplification	Broad	-	-	-	-	Amplification	Focal	CCNE
