# Supplementary material for: Genome-Wide Identification of Somatic Aberrations from Paired Normal-Tumor Samples
Source: PLoS One. 2014 Jan 30;9(1):e87212. doi: 10.1371/journal.pone.0087212 (PMC3907544; doi:10.1371/journal.pone.0087212)
Supplement: File S1 — contains the following files. Text S1. Detailed description of statistical methods in GIANT. Figure S1. Comparison of BAF signals in two normal replicates on different chromosomes. Figure S2. Visualized genomic aberrations for replicate pairs. Figure S3. Comparison of estimated normal cell contamination levels and tumor average copy number for simulated tumor SNP arrays. Figure S4. Comparison of specificity for identifying normal regions in simulated tumor samples. Figure S5 Sensitivities for distinguishing aberrations from normal regions by five different methods. Figure S6. Theoretical and real mean values of LRR/BAF in different scenarios. Figure S7. Aberrations in chromosome 6 of breast cancer cell line CRL-2324. Figure S8. Aberrations in chromosome 13 of breast cancer cell line CRL-2324. Figure S9. Comparison of results for breast cancer cell line CRL-2324 by different methods. Figure S10. Comparison of self-consistency for GIANT and GPHMM using dilution series data. Figure S11. Visualized results of GIANT for replicate breast cancer sample. Figure S12. Detection of germline homozygous deletion of ADAM3A gene in diluted breast tumor sample 7207. Figure S13. Comparison of the results for chromosome 8 of breast tumor 7207 by different methods. Figure S14. Significance test of genome-wide somatic amplification and LOH for urothelial cancer samples. Figure S15. Significance test of genome-wide somatic deletion for urothelial cancer samples. Figure S16. Box plots of summarized results for 112 breast cancer samples. Figure S17. Distinct patterns of genome-wide copy number profiles identified in two groups of tumors. Figure S18. Genome-wide CNA profiles of breast cancer samples. Figure S19. Frequency plot of genomic-wide CNA for 112 breast cancer samples. Figure S20. Frequency plot of genomic-wide LOH for 112 breast cancer samples. Table S1. Detailed information of hidden states in PSHMM. Table S2. Parameters estimated by GIANT on replicate SNP arrays. Table S [file pone.0087212.s001.zip › TextS1.pdf]

Genome-wide identification of somatic aberrations from normal-tumor samples

Supplementary material

Content

Computational identification of somatic copy number aberrations and loss of heterozygosity from tumor and paired normal samples 1
1. Hidden Markov Model for Paired SNP-array data (PSHMM) 3
1.1. Introduction 3
1.2. Hidden States 4
1.3. Emission models 4
1.4. Transition matrix and initial state distribution 6
1.5. EM algorithm and parameter update 7
2. Test of Statistical significance for tumor aberrations 10

1. Hidden Markov Model for Paired SNP-array data (PSHMM)

1.1. Introduction

For SNP-arrays, the genotyping signals include probe intensities presenting abundance of A and B alleles at each interrogated SNP, respectively. In practice, genotyping signals are transformed into two different measurements: Log R Ratio (LRR) and B Allele Frequency (BAF). The LRR measurement represents total probe intensity and is associated with the copy number of both A and B alleles, whilst BAF reflects the proportion of B allele at each probe, described as the ratio of the probe intensity of B allele to the total probe intensity. More detailed information regarding the definition and calculation of LRR and BAF measurements from raw genotyping signals can be found in for Illumina platform and for Affymetrix platform[1, 2].

From the SNP-array data of normal DNA sample, calling copy number variation at each SNP (CNV) is a relatively easy task. Many approaches, such as PennCNV [3], have been demonstrated as efficient tools for such studies. However, it is much more challenging to accurately identify somatic genomic aberrations, such as copy number alterations (CNA) and loss of heterozygosity (LOH), from SNP-array data of tumor sample, mainly because tumor sample usually includes not only cancer but also normal cells [4-15].

Generally, suppose there are two samples collected in the study: one is a mixed sample consisting of two kinds of genetically related cells (denoted as c_1, c_2) with different genotypes, and the other is a paired sample consisting of only c_2. SNP-array experiments are performed on both samples rendering a pair of genotyping data. The genotype of c_2 can be explicitly determined from SNP-array data of the paired sample, but the genotype of c_2 is "hidden" as the genotyping signal of the mixed sample is actually generated from a mixture of DNA from both c_1 and c_2 with unknown proportion. So the goal is to determine c_1's genotype information (represented by total copy number contributed by both alleles and proportion of B allele, denoted as $n_{i,c1}$ $u_{i,c_1}(i=1,...,n)$) and corresponding genomic aberrations from the SNP-array data of the mixed sample with the aid of genotype information of c_2 inferred from SNP-array of the paired sample. Note that the genotype of c_2 is obtainable by analyzing the SNP-array data of the paired pure sample using available tools such as GPHMM[6],

therefore by using the genotyping information, we can uniquely determine the corresponding total copy number contributed by both alleles and proportion of B allele, denoted as n_{i,c_2} and u_{i,c_2} $(i=1,...,n)$ respectively. It should be pointed out that with the existence of germline aberration, c_2 is not restricted to diploid genotypes AA, AB or BB. Instead, it can be any possible genotype associated with different genomic variations described in Table S1.

1.2. Hidden States

Table S1 shows the definitions of the hidden states used in PSHMM, including different kinds of genomic abnormalities such as copy number gain/ loss and LOH. Due to saturation effects in array hybridization, hidden states are limited to aberrations with copy number less than 8 to ensure they are discriminable by the genotyping signals.

1.3. Emission models

One important step toward profiling c_1's genomic aberration from mixed genotyping signal in SNP-array experiment is to determine the proportion of c_2 in the mixed sample. Some computational methods [6, 11, 14, 15] have been proposed to automatically infer the proportion of cancer cells by de-convoluting the genotyping signals generated from mixed sample DNAs. In PSHMM, we adopted empirical formulas proposed by SiDCoN [16], in which relationship of LRR/BAF measurements and cancer cell proportion in mixed sample is quantitatively modeled. Specifically, let the LRR and BAF of the ith probe be l_i and b_i $(i=1,...,n)$, then their expectations can be formulated as follow:

$$E(l_i) = \log_{10}((wn_{i,c1} + (1-w)n_{i,c2})/2) \tag{1}$$

$$E(b_i) = \frac{wn_{i,c1}u_{i,c1} + (1-w)n_{i,c2}u_{i,c2}}{wn_{i,c1} + (1-w)n_{i,c2}} \tag{2}$$

where w is the proportion of cancer cells in the mixed sample.

On the other hand, owning to the complicated characteristic of tumor, genotyping signals of SNP-array are affected by other factors in practice, such as tumor aneuploidy [6, 11, 12, 14, 15] and genomic waves related to local GC contents [17]. We further use following formula to take all these effects into account.

$$E(l_i) = \log_{10}((wn_{i,c1} + (1-w)n_{i,c2})/2 + hg_i + o) \tag{3}$$

where g_i is the local GC percentage at the ith probe and h is the coefficient. o is the shift of the LRR baseline. By assuming genotyping signals are normally distributed, we can then formulate the emission probabilities of each observed (l_i, b_i) given hidden state s(s>1) and k^{th} genotype of c_1:

$$f_p(l_i \mid w,h,o,\sigma_l,s,n_{i,c2}) = \frac{1}{\sigma_l}\phi\left(\frac{l_i - (2\log_{10}(y_{i,p}(s)/2)+o+hg_i)}{\sigma_l}\right) \tag{4}$$

$$f_p(b_i \mid w,\sigma_b,k,s,n_{i,c2},u_{i,c2}) = \sum_{k=1}^{G}\frac{1}{\sigma_b}p_i(k \mid s)\phi\left(\frac{b_i - z_{i,p}(k,s)/y_{i,p}(s)}{\sigma_b}\right) \tag{5}$$

with

$$y_{i,p}(s) = wn_{i,c1}(s) + (1-w)n_{i,c2} \tag{6}$$

$$z_{i,p}(k,s) = wn_{i,c1}u_{i,c1}(k,s) + (1-w)n_{i,c2}u_{i,c2} \tag{7}$$

where σ_l, σ_b denote the variance of LRR and BAF, respectively. G is the number of genotypes in state s. $p_i(k \mid s)$ is the prior probability of observing k^{th} genotype (conditional on s) at the ith probe in the mixed sample by splitting the probabilities of homozygous and heterozygous genotypes estimated from the allelic frequencies in population. Note that the genotypes of c_1 and c_2 are genetically related, therefore restrictions are imposed on equation (4) and (5). For example, if c_2's genotype is 'AA' then we need to rule out the possibility that c_2 has genotype 'ABB', since a homozygous genotype cannot turn into a heterozygous genotype during somatic aberrations.

We incorporate the effect of signal fluctuation in the emission models of PSHMM, and in this case a uniform distribution is employed to approximate the statistical distributions of LRR and BAF:

$$f_l(l_i) = \begin{cases} \frac{1}{(b-a)}, a \le l_i \le b \\ 0, \ otherwise \end{cases} \tag{8}$$

$$f_l(b_i) = \begin{cases} \frac{1}{(b'-a')}, a' \le b_i \le b' \\ 0, \ otherwise \end{cases} \tag{9}$$

Note that genotyping error also happens in the SNP-array of the paired sample,

rendering wrong identification of genotypes in c_2, therefore equation (4) and (5) should be replaced with the emission models used for single SNP-array analysis [6], in which the genotypes of c_2 are limited to normal by assuming there is no genomic variant:

$$f_e(l_i \mid w,h,o,\sigma_l,s) = \frac{1}{\sigma_l}\phi\left(\frac{l_i - (2\log_{10}(y_{i,e}(s)/2)+o+hg_i)}{\sigma_l}\right) \tag{10}$$

$$f_e(b_i \mid w,\sigma_b,k,s) = \sum_{k=1}^{G}\frac{1}{\sigma_b}p_i(k,s)\phi\left(\frac{b_i - z_{i,e}(k,s)/y_{i,e}(s)}{\sigma_b}\right) \tag{11}$$

with

$$y_{i,e}(s) = 2+(n_{i,s}-2)w \tag{12}$$

$$z_{i,e}(k,s) = 2u_{i,c2}+(n_{i,s}u_{i,k,s}-2u_{i,c2})w \tag{13}$$

Taken together, the emission probability of PSHMM is formulated as the total probability:

$$\begin{aligned} &f(l_i,b_i \mid w,h,o,\sigma_l,\sigma_b,k,s,n_{i,c2},u_{i,c2}) = \\ &\quad (1-p_{i,l})[(1-p_{i,e})f_p(l_i \mid w,h,o,\sigma_l,s,n_{i,c2})f_p(b_i \mid w,\sigma_b,k,s,,n_{i,c2},u_{i,c2}) \\ &\quad +p_{i,e}f_e(l_i \mid w,h,o,\sigma_l,s)f_e(b_i \mid w,\sigma_b,k,s)]+p_{i,l}f_l(l_i)f_l(b_i) \end{aligned} \tag{14}$$

where $p_{i,e}$ is the probability of genotype errors in paired sample for the ith probe (default value is 0.01). $p_{i,l}$ is the probability of observing genotyping signal fluctuation in the mixed sample for the ith probe (default value is 0.01).

1.4. Transition matrix and initial state distribution

In PSHMM, we use a transition matrix similar with that adopted in our previous work [6], associated with an ergodic Markov chain with initial transition matrix A^0 defined as follow:

$$A_{kl}^{(0)} = \begin{cases} p_t/(M-1), & k \neq l \\ 1-p_t, & k = l \end{cases}, \quad k,l = 1,2,\ldots,M \tag{15}$$

where $A_{kl}^{(0)}$ indicates the element of the kth row and l^{th} column in the initial transition matrix, p_t is the initial probability of transitions from state i to any other states (default value is 10^{-5}). M is the number of all hidden state. By assuming there is no additional information of the hidden states, the prior probabilities of initial states are then:

$$\pi_k^{(0)} = 1/N, \quad k = 1,2,\ldots,M \tag{16}$$

1.5. EM algorithm for parameter estimation

We use Expectation Maximization (EM) algorithm [18] for HMM training and parameter estimation in this study, which is aimed to find maximum likelihood estimates of parameters associated with hidden latent variables. The EM algorithm consists of an expectation (E) step and a maximization (M) step. During the E step, the expectation of the log-likelihood is calculated by using the current estimate for the parameters, whilst during the M step, parameters are updated by maximizing the expected log-likelihood generated in the E step. These two steps are performed iteratively until the algorithm converges to a local maxima or reaches to a pre-defined threshold for maximal number of iterations. In addition, as we previously proposed [6], a grid search of parameters o and w is performed in PSHMM in order to find optimal initial values.

To update the elements of state transition matrix, we adopt the standard approach of HMM modeling:

$$A_{kl}^{(n)} = \frac{\sum_{i=1}^{N-1} \xi_i^{(n)}(k,l)}{\sum_{i=1}^{N-1} \gamma_i^{(n)}(k)} \tag{17}$$

where $\xi_i^{(n)}(k,l)$ is the probability of state transition from k (probe i) to l (probe i+1) calculated the nth iteration, given all observed data and the HMM model with parameters calculated in the n-1th iteration. Similarly, $\gamma_i^{(n)}(k)$ is the probability of state k at the probe i in the nth iteration. Both $\xi_i^{(n)}(k,l)$ and $\gamma_i^{(n)}(k)$ are obtainable using the forward-backward algorithm and more detailed information is provided in [18]. Also the probabilities of initial states are updated as follow:

$$\pi_k^{(n)} = \gamma_1^{(n)}(k) \tag{18}$$

Furthermore, to estimate parameters in emission probability such as o and σ_l, we need to reformulate the EM algorithm. Given LRR and BAF of the mixed sample, as the E step we first calculate the partial log-likelihood containing emission probability functions [18] as follow:

$$LL = \sum_{i=1}^{N} \sum_{s=1}^{M} \sum_{k=1}^{N_g} I_i(s) \log(f(l_i, b_i \mid w, h, o, \sigma_l, \sigma_b, k, s, n_{i,c2}, u_{i,c2})) \tag{19}$$

where $I_i(s)$ is an indicator function with value 1 if the ith SNP is in state s, otherwise

it is set to 0. Then the expectation of the partial log-likelihood is then formulated as:

$$E(LL)=\sum_{i=1}^{N}\sum_{s=1}^{M}\gamma_{i,p}^{(n)}(s)\{\log(f_p(l_i\,|\,w,h,o,\sigma_l,s,n_{i,c2}))+\log(f_p(b_i\,|\,w,\sigma_b,k,s,n_{i,c2},u_{i,c2}))\}$$
$$+\gamma_{i,e}^{(n)}(s)\{\log(f_e(l_i\,|\,w,h,o,\sigma_l,s))+\log(f_e(b_i\,|\,w,\sigma_b,k,s))\}+(1-\gamma_{i,p}^{(n)}(s)-\gamma_{i,e}^{(n)}(s))$$
$$\{\log(f_l(l_i))+\log(f_l(b_i))\} \tag{20}$$

where $\gamma_{i,p}^{(n)}(s)$ and $\gamma_{i,e}^{(n)}(s)$ are the conditional probabilities that equation (4-5) and (9-10) hold respectively, given state *s* at the probe *i* in the nth iteration, which can be calculated by the following equations:

$$\gamma_{i,p}^{(n)}(s)=\gamma_i^{(n)}(s)(1-p_{i,e}^{'}-p_{i,f}^{'}) \tag{21}$$

$$\gamma_{i,e}^{(n)}(s)=\gamma_i^{(n)}(s)(1-p_{i,e}^{'}) \tag{22}$$

where $p_{i,e}^{'}$ and $p_{i,f}^{'}$ indicate probabilities of genotyping error and fluctuation given all aforementioned parameters and data:

$$p_{i,e}^{'}=\frac{(1-p_{i,l})p_{i,e}f_e(l_i\,|\,w,h,o,\sigma_l,s)f_e(b_i\,|\,w,\sigma_b,k,s)}{f(l_i,b_i\,|\,w,h,o,\sigma_l,\sigma_b,k,s,n_{i,c2},u_{i,c2})} \tag{23}$$

$$p_{i,l}^{'}=\frac{p_{i,l}f_l(l_i)f_l(b_i)}{f(l_i,b_i\,|\,w,h,o,\sigma_l,\sigma_b,k,s,n_{i,c2},u_{i,c2})} \tag{24}$$

Next, in order to derive the formula for updating *o* in the M step, we take the partial derivative with respect to *o*:

$$\frac{\partial E(LL)}{\partial o}=0 \tag{25}$$

By solving above equation, we obtain the formula as follow, in which the values of all other parameters are determined in previous iteration.

$$o^{(n)}=\frac{\sum_{i=1}^{N}\sum_{s=1}^{M}\gamma_{i,p}^{(n)}(s)g_i[l_i-h^{(n-1)}g_i-2\log_{10}(y_{i,p}^{(n)}(s)/2)]+\gamma_{i,e}^{(n)}(s)g_i[l_i-h^{(n-1)}g_i-2\log_{10}(y_{i,e}^{(n)}(s)/2)]}{\sum_{i=1}^{N}\sum_{s=1}^{M}\gamma_{i,p}^{(n)}(s)+\gamma_{i,e}^{(n)}(s)} \tag{26}$$

Similarly, we use following formulas to update the remaining parameters appeared in the emission model consecutively. Note that once the value of a parameter is changed in the nth iteration, it will be then used for the following updating procedure of other

parameters.

$$h^{(n)} = \frac{\sum_{i=1}^{N}\sum_{s=1}^{M} \gamma_{i,p}^{(n)}(s) g_i [l_i - o^{(n)} - 2\log_{10}(y_{i,p}^{(n)}(s)/2)] + \gamma_{i,e}^{(n)}(s) g_i [l_i - o^{(n)} - 2\log_{10}(y_{i,e}^{(n)}(s)/2)]}{\sum_{i=1}^{N}\sum_{s=1}^{M} (\gamma_{i,p}^{(n)}(s) + \gamma_{i,e}^{(n)}(s)) g_i^2} \tag{27}$$

$$\sigma_l^{(n)} = \left[\frac{\sum_{i=1}^{N}\sum_{s=1}^{M} \gamma_{i,p}^{(n)}(s)(l_i - o^{(n)} - h^{(n)} g_i - 2\log_{10}(y_{i,p}^{(n)}(s)/2))^2}{\sum_{i=1}^{N}\sum_{s=1}^{M} \gamma_{i,p}^{(n)}(s) + \gamma_{i,e}^{(n)}(s)} + \frac{\sum_{i=1}^{N}\sum_{s=1}^{M} \gamma_{i,e}^{(n)}(s)(l_i - o^{(n)} - h^{(n)} g_i - 2\log_{10}(y_{i,e}^{(n)}(s)/2))^2}{\sum_{i=1}^{N}\sum_{s=1}^{M} \gamma_{i,p}^{(n)}(s) + \gamma_{i,e}^{(n)}(s)}\right]^{\frac{1}{2}} \tag{28}$$

$$\sigma_b^{(n)} = \left[\frac{\sum_{i=1}^{N}\sum_{s=1}^{M} \gamma_{i,p}^{(n)}(s) \sum_{k=1}^{G} p_i(k|s)(b_i - \frac{z_{i,p}^{(n)}(k,s)}{y_{i,p}^{(n)}(k,s)})^2 + \sum_{i=1}^{N}\sum_{s=1}^{M} \gamma_{i,e}^{(n)}(s) \sum_{k=1}^{G} p_i(k|s)(b_i - \frac{z_{i,e}^{(n)}(k,s)}{y_{i,e}^{(n)}(k,s)})^2}{\sum_{i=1}^{N}\sum_{s=1}^{M} \gamma_{i,p}^{(n)}(s) + \gamma_{i,e}^{(n)}(s)}\right]^{\frac{1}{2}} \tag{29}$$

with

$$y_{i,p}^{(n)}(s) = w^{(n-1)} n_{i,s} + (1 - w^{(n-1)}) n_{i,c2} \tag{30}$$

$$z_{i,p}^{(n)}(k,s) = w^{(n-1)} n_{i,s} u_{i,k,s} + (1 - w^{(n-1)}) n_{i,c2} u_{i,c2} \tag{31}$$

$$y_{i,e}^{(n)}(s) = 2 + (n_{i,s} - 2) w^{(n-1)} \tag{32}$$

$$z_{i,e}^{(n)}(k,s) = 2u_{i,c2} + (n_{i,s} u_{i,k,s} - 2u_{i,c2}) w^{(n-1)} \tag{33}$$

Unfortunately, there is no close-form solution for equation:

$$\frac{\partial E(LL)}{\partial w} = 0 \tag{34}$$

therefore we use the Newton-Raphson method to numerically increase the expectation of the log-likelihood, and update w using the following formula:

$$w^{(n)} = w^{(n-1)} - \frac{\frac{\partial E(LL)}{\partial w}}{\frac{\partial^2 E(LL)}{\partial^2 w}} \tag{35}$$

2. Test of Statistical significance for tumor aberrations

Somatic aberrations in cancer genome can be classified as two subtypes: passenger and driver aberration, and the latter is associated with tumorigenesis due to its functional importance. A statistical approach has been introduced in GISTIC [19, 20] to discover driver aberrations that are statistically significant in multiple tumor samples across whole genome. However, one shortcoming of this approach is that it directly uses raw affymetrix SNP-array data and therefore is prone to critical issues that can largely affect the raw genotyping signals, such as normal cell contamination, signal baseline shift and signal noise by GC content. To solve this problem, we propose to use the genome-wide somatic aberration profiles generated by GIANT for pinpointing driver aberrations, which can efficiently avoid aforementioned issues.

Suppose there are totally M tumor samples and each consists of N probes along the genome. For amplified aberrations, we used statistic G_i^{amp} to reflect the amplitude and frequency of an amplified region in the aberration profiles of these tumor samples, which is defined as follow:

$$G_i^{amp} = \sum_{j=1}^{M} \max(C_{ij}^T - C_{ij}^N, 0) I(x_{ij}^N) \tag{36}$$

where C_{ij}^{T} and $C_i^{(\mathrm{N}}$ are the copy numbers for the ith probe in tumor sample j and its matched normal sample, respectively. To make sure the aberration is indeed somatic, we require that the statistic only accounts for aberration regions where there is no germline mutation in the corresponding genomic region of the normal sample. It is implemented by indicator function $\mathrm{I}(x_{ij}^{\mathrm{N}})$, which returns 0 if the inferred state x_{ij}^{N} for probe i of normal sample j is the normal state and 1 otherwise. Similarly, for deleted regions the statistic G_i^{del} is calculated as below:

$$G_i^{del} = \sum_{j=1}^{M} \max(C_{ij}^N - C_{ij}^T, 0) I(x_{ij}^N) \tag{37}$$

On the other hand, the statistic for LOH regions $\mathrm{G_i^{LOH}}$ is defined as below

$$G_i^{LOH} = \sum_{j=1}^{M} L_{ij}^T I(x_{ij}^N) \tag{38}$$

where L_{ij}^{T} is the inferred LOH state in tumor sample.

To get the empirical distribution of statistic G_i under the null hypothesis that the pinpointed region is not driver but passenger mutation, we adopted the procedure introduced in [19] by which the exact estimation of the null distribution can be efficiently obtained by assuming the sum of the statistic across all samples equals the convolution of the statistic in each tumor sample. Specifically, let h_i be the histogram of the statistics in the ith sample obtained by above equations. The exact distribution H of statistic G_i can be obtained by calculating their convolutions:

$$\mathrm{H} = \mathrm{h}_1 \otimes \mathrm{h}_2 \otimes \ldots \otimes \mathrm{h}_N \quad (39)$$

Based on the null distribution, we can estimate the statistical significance of each aberration by measuring the associated p-value using statistic G_i. In addition, to diminish the rate of the type I errors in multiple hypothesis testing, we made further corrections by adopting the q-values from FDR. The q-value threshold of 0.25 used in GISTIC was adopted in this study. If an aberration has an associated q-value less than this threshold, it will be determined as significant driver mutation.

Reference

1. Curtis C, Lynch AG, Dunning MJ, Spiteri I, Marioni JC, Hadfield J, Chin SF, Brenton JD, Tavare S, Caldas C: **The pitfalls of platform comparison: DNA copy number array technologies assessed.** *BMC Genomics* 2009, **10:**588.
2. Peiffer DA, Le JM, Steemers FJ, Chang W, Jenniges T, Garcia F, Haden K, Li J, Shaw CA, Belmont J, et al: **High-resolution genomic profiling of chromosomal aberrations using Infinium whole-genome genotyping.** *Genome Res* 2006, **16:**1136-1148.
3. Wang K, Li M, Hadley D, Liu R, Glessner J, Grant SF, Hakonarson H, Bucan M: **PennCNV: an integrated hidden Markov model designed for high-resolution copy number variation detection in whole-genome SNP genotyping data.** *Genome Res* 2007, **17:**1665-1674.
4. Assie G, LaFramboise T, Platzer P, Bertherat J, Stratakis CA, Eng C: **SNP arrays in heterogeneous tissue: highly accurate collection of both germline and somatic genetic information from unpaired single tumor samples.** *Am J Hum Genet* 2008, **82:**903-915.
5. Greenman CD, Bignell G, Butler A, Edkins S, Hinton J, Beare D, Swamy S, Santarius T, Chen L, Widaa S, et al: **PICNIC: an algorithm to predict absolute allelic copy number variation with microarray cancer data.** *Biostatistics* 2010, **11:**164-175.
6. Li A, Liu Z, Lezon-Geyda K, Sarkar S, Lannin D, Schulz V, Krop I, Winer E, Harris L, Tuck D: **GPHMM: an integrated hidden Markov model for identification of copy number alteration and loss of heterozygosity in complex tumor samples using whole genome SNP arrays.** *Nucleic Acids Res* 2011, **39:**4928-4941.

7. Liu Z, Li A, Schulz V, Chen M, Tuck D: **MixHMM: inferring copy number variation and allelic imbalance using SNP arrays and tumor samples mixed with stromal cells.** *PLoS One* 2010, **5:**e10909.
8. Nilsen G, Liestol K, Van Loo P, Moen Vollan HK, Eide MB, Rueda OM, Chin SF, Russell R, Baumbusch LO, Caldas C, et al: **Copynumber: Efficient algorithms for single- and multi-track copy number segmentation.** *BMC Genomics* 2012, **13:**591.
9. Olshen AB, Bengtsson H, Neuvial P, Spellman PT, Olshen RA, Seshan VE: **Parent-specific copy number in paired tumor-normal studies using circular binary segmentation.** *Bioinformatics* 2011, **27:**2038-2046.
10. Ortiz-Estevez M, Aramburu A, Bengtsson H, Neuvial P, Rubio A: **CalMaTe: a method and software to improve allele-specific copy number of SNP arrays for downstream segmentation.** *Bioinformatics* 2012, **28:**1793-1794.
11. Popova T, Manie E, Stoppa-Lyonnet D, Rigaill G, Barillot E, Stern MH: **Genome Alteration Print (GAP): a tool to visualize and mine complex cancer genomic profiles obtained by SNP arrays.** *Genome Biol* 2009, **10:**R128.
12. Rasmussen M, Sundstrom M, Goransson Kultima H, Botling J, Micke P, Birgisson H, Glimelius B, Isaksson A: **Allele-specific copy number analysis of tumor samples with aneuploidy and tumor heterogeneity.** *Genome Biol* 2011, **12:**R108.
13. Staaf J, Lindgren D, Vallon-Christersson J, Isaksson A, Goransson H, Juliusson G, Rosenquist R, Hoglund M, Borg A, Ringner M: **Segmentation-based detection of allelic imbalance and loss-of-heterozygosity in cancer cells using whole genome SNP arrays.** *Genome Biol* 2008, **9:**R136.
14. Van Loo P, Nordgard SH, Lingjaerde OC, Russnes HG, Rye IH, Sun W, Weigman VJ, Marynen P, Zetterberg A, Naume B, et al: **Allele-specific copy number analysis of tumors.** *Proc Natl Acad Sci U S A* 2010, **107:**16910-16915.
15. Yau C, Mouradov D, Jorissen RN, Colella S, Mirza G, Steers G, Harris A, Ragoussis J, Sieber O, Holmes CC: **A statistical approach for detecting genomic aberrations in heterogeneous tumor samples from single nucleotide polymorphism genotyping data.** *Genome Biol* 2010, **11:**R92.
16. Nancarrow DJ, Handoko HY, Stark MS, Whiteman DC, Hayward NK: **SiDCoN: a tool to aid scoring of DNA copy number changes in SNP chip data.** *PLoS One* 2007, **2:**e1093.
17. Diskin SJ, Li M, Hou C, Yang S, Glessner J, Hakonarson H, Bucan M, Maris JM, Wang K: **Adjustment of genomic waves in signal intensities from whole-genome SNP genotyping platforms.** *Nucleic Acids Res* 2008, **36:**e126.
18. Rabiner LR: **A Tutorial on Hidden Markov-Models and Selected Applications in Speech Recognition.** *Proceedings of the Ieee* 1989, **77:**257-286.
19. Beroukhim R, Getz G, Nghiemphu L, Barretina J, Hsueh T, Linhart D, Vivanco I, Lee JC, Huang JH, Alexander S, et al: **Assessing the significance of chromosomal aberrations in cancer: methodology and application to glioma.** *Proc Natl Acad Sci U S A* 2007, **104:**20007-20012.
20. Mermel CH, Schumacher SE, Hill B, Meyerson ML, Beroukhim R, Getz G: **GISTIC2.0 facilitates sensitive and confident localization of the targets of focal somatic copy-number alteration in human cancers.** *Genome Biol* 2011, **12:**R41.
